# Analysis of Melatonin Secretion Patterns in Buffalo Milk and Its Association with *AANAT* Polymorphisms

**DOI:** 10.3390/ijms27209178

**Published:** 2026-10-16

**Authors:** Songyuan You, Yiheng Fu, Yanghua Li, Jiaqi Jiang, Eng Huan Hau, Xingqi Wei, Zhixiang Long, Qingkun Zeng, Qingyou Liu, Ling Li

**Affiliations:** 1 Guangxi Zhuang Autonomous Region Buffalo Milk Quality and Safety Control Technology Engineering Research Center, Guangxi Buffalo Research Institute, Nanning 530001, China; 2 Guangxi Key Laboratory of Animal Reproduction, Breeding and Disease Control, College of Animal Science and Technology, Guangxi University, Nanning 530004, China; 3 Guangxi Zhuang Autonomus Region Center for Analysis and Test Research, Nanning 530022, China; 4 College of Animal Science, South China Agricultural University, Guangzhou 510640, China

**Keywords:** buffalo milk, melatonin, secretion pattern, *AANAT*, SNP

## Abstract

Melatonin (MT) is an important bioactive functional component of milk, where its content and biosynthetic mechanism are of considerable relevance to the quality regulation of dairy buffalo milk. This study aimed to investigate the variation patterns and biosynthetic mechanisms of melatonin in milk from dairy buffaloes. A total of 125 buffalo milk samples were collected, and MT content was determined using liquid chromatography–tandem mass spectrometry (LC–MS/MS). MT secretion patterns were evaluated according to breed, lactation, parity, and milking time (light exposure). In addition, whole-genome sequencing (WGS) data were used to screen for single-nucleotide polymorphisms (SNPs), which are significantly associated with melatonin biosynthetic enzyme genes. The results showed that breed, milking time, and lactation were the major factors affecting MT content in buffalo milk. Based on the WGS data, two SNPs significantly associated with MT secretion and synthesis were identified within the aralkylamine N-acetyltransferase (*AANAT*) region, among which the GG genotype at 3-8019407 and the CC genotype at 3-8019446 were associated with higher MT content. The MT content for both wild-type homozygotes was 59.54 pg/mL, which was higher than that of heterozygotes (39.12 pg/mL) (*p* < 0.05). With the exception of SNP2 and SNP8, all other SNPs were in Hardy–Weinberg equilibrium. These findings will serve as a reference for the subsequent selection and identification of breeding materials for high MT dairy buffalo, as well as for the development of high MT buffalo milk products.

## 1. Introduction

Melatonin (MT) is a kind of bioactive indoleamine molecule derived from tryptophan metabolism, which was first isolated and identified from the bovine pineal gland [1]. Apart from the pineal gland, melatonin is also synthesized in organs such as the gastrointestinal tract, liver, kidney, mammary glands, reproductive tissue and mitochondria, and participates in many physiological processes of the body [2,3,4,5]. The synthesis of MT in mammals is typically based on tryptophan as a precursor, and it is gradually converted by the 5-hydroxytryptamine (serotonin) pathway [6], in which arylalkylamine N-acetyltransferase (*AANAT*) and acetylserotonin O-methyltransferase (*ASMT*) are the key rate-limiting enzymes in this pathway. These enzymes play an important role in the regulation of MT synthesis [7,8]. *AANAT* activity and its expression level have obvious circadian rhythm characteristics, typically increasing at night and being significantly influenced by light signals. Hence, it is considered to be an important regulatory gene that connects the external photoperiod changes with the body‘s melatonin secretion rhythm [9,10].

Milk is an important source of nutrition for young animals and humans, containing a variety of bioactive substances with regulatory functions [11]. MT in milk has attracted much attention due to its potential nutritional value and functional milk development prospects [12]. Studies have shown that the content of MT in milk is affected by many factors such as light time, light intensity, and management methods, with milk samples collected at night or under low-light conditions typically containing higher levels of MT [11,12,13,14]. Currently, the primary approach involves supplementing rumen-protected tryptophan or exogenous melatonin to increase melatonin content in milk or blood; meanwhile, elevated MT content can effectively improve milk quality and reduce somatic cell counts in milk [15,16], which indicates that there may be a close relationship between melatonin, teat health and milk quality formation.

Buffalo is an important dual-purpose livestock resource in southern China. Its milk has excellent characteristics, such as high milk fat, high protein and high calcium [17], and contains significantly higher magnesium content than cow’s milk [18]. This makes it highly valuable for the processing of specialty dairy products and the development of functional dairy products. Hence, conducting research on the genetic basis of buffalo milk quality traits is of great significance for improving local buffalo germplasm resources and promoting the development of the buffalo industry. In recent years, whole-genome sequencing (WGS), SNP genotyping and marker-assisted selection (MAS) have been gradually applied for buffalo genetic diversity analysis [19], candidate gene mining of reproductive traits [20], disease diagnosis [21], and functional dairy product development [22], providing important means for elucidating the genetic mechanisms underlying complex traits.

In recent years, research on the regulation mechanism of MT in milk has mainly focused on species such as cattle, goats and sheep. For example, in Holstein cows, polymorphisms in the *AANAT* and *ASMT* have been shown to be significantly associated with melatonin content in milk and peripheral blood. Some SNPs can be used as potential molecular markers for screening individuals with high MT milk [23]. Additionally, previous BSA analysis of dairy goats further showed that SNPs of *ASMT* and MT receptor-related genes were related to MT in milk and serum [24]. These association findings are further supported by relevant studies: the generation of *AANAT*/*ASMT* transgenic sheep or engineering overexpression of *ASMT* in goats using CRISPR/Cas9, which can upregulate the expression, increase the content of MT in milk, and improve the milk quality and anti-inflammatory capacity of sheep milk [25,26]. These studies have shown that the difference in MT secretion in milk of different individuals may be related to the genetic variation of MT synthesis-related genes. However, there has been a lack of systematic research on the variation of MT content in buffalo milk and its relationship with *AANAT* polymorphism. If the correlation between buffalo milk melatonin variation and *AANAT* polymorphism can be clarified, valuable evidence will be provided for exploring the genetic mechanism underlying milk MT synthesis in buffalo.

Therefore, this study systematically analyzed the secretion of MT in buffalo milk from different varieties, as well as different lactation, parity and milking time using 125 buffalo milk samples. The SNPs significantly related to the synthesis and secretion of MT in milk in the *AANAT* region were screened using whole-genome sequencing data, and the correlation between different genotypes and MT content was analyzed.

## 2. Results

### 2.1. Distribution of MT Content in 125 Buffalo Milk Samples

**Figure 1:**
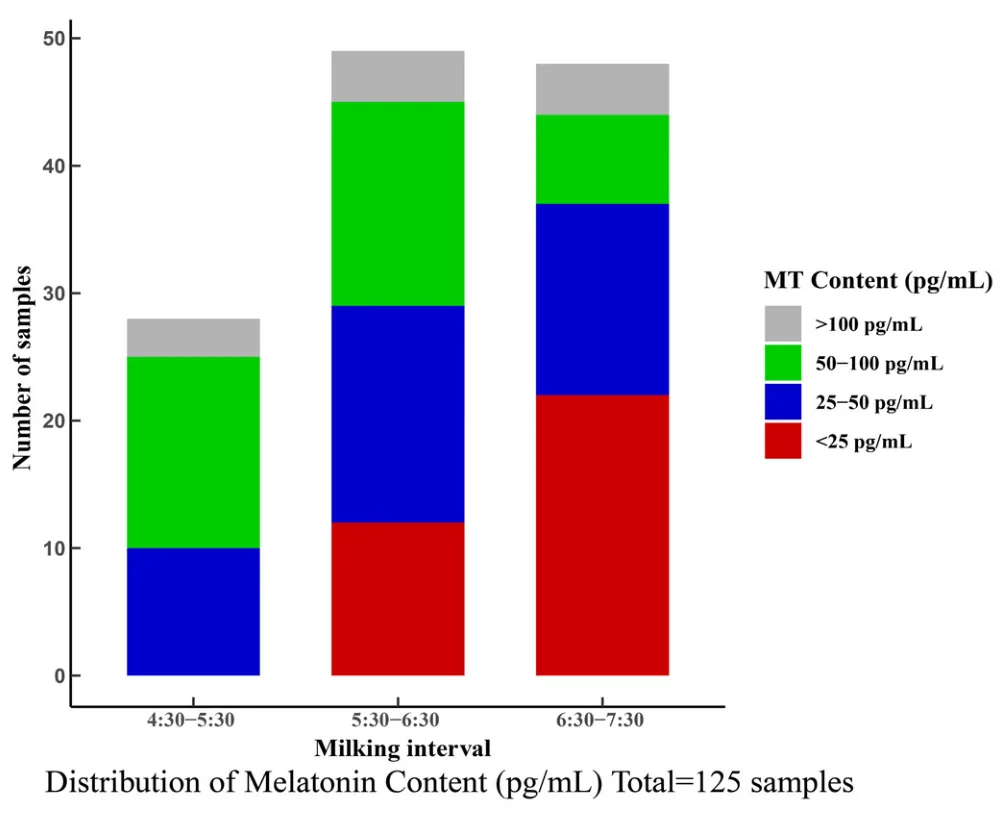
Distribution of MT content in 125 buffalo milk samples.

A total of 125 buffalo milk samples (125 buffalo individuals, one sample per individual) were continuously collected from the Buffalo Breeding Farm in Guangxi Zhuang Autonomous Region within a unified experimental period. Descriptive statistics were performed to characterize the distribution of MT content in 125 buffalo milk samples. The results revealed obvious individual differences in milk melatonin (MT) content among dairy buffaloes, with the values ranging from 0.80 to 132.67 pg/mL. As shown in Figure 1, there were 28 samples from 4:30–5:30 am, 49 samples from 5:30–6:30 am, and 48 samples from 6:30–7:30 am. Among these samples, 34 milk samples had levels below 25 pg/mL, accounting for 27.20%; 42 milk samples had levels between 25 and 50 pg/mL, accounting for 33.60%; 38 milk samples had levels between 50 and 100 pg/mL, accounting for 30.40%; and 11 milk samples had levels above 100 pg/mL, accounting for 8.80%.

### 2.2. Analysis of Factors Influencing MT Content and Secretion Patterns in Buffalo Milk

**Figure 2:**
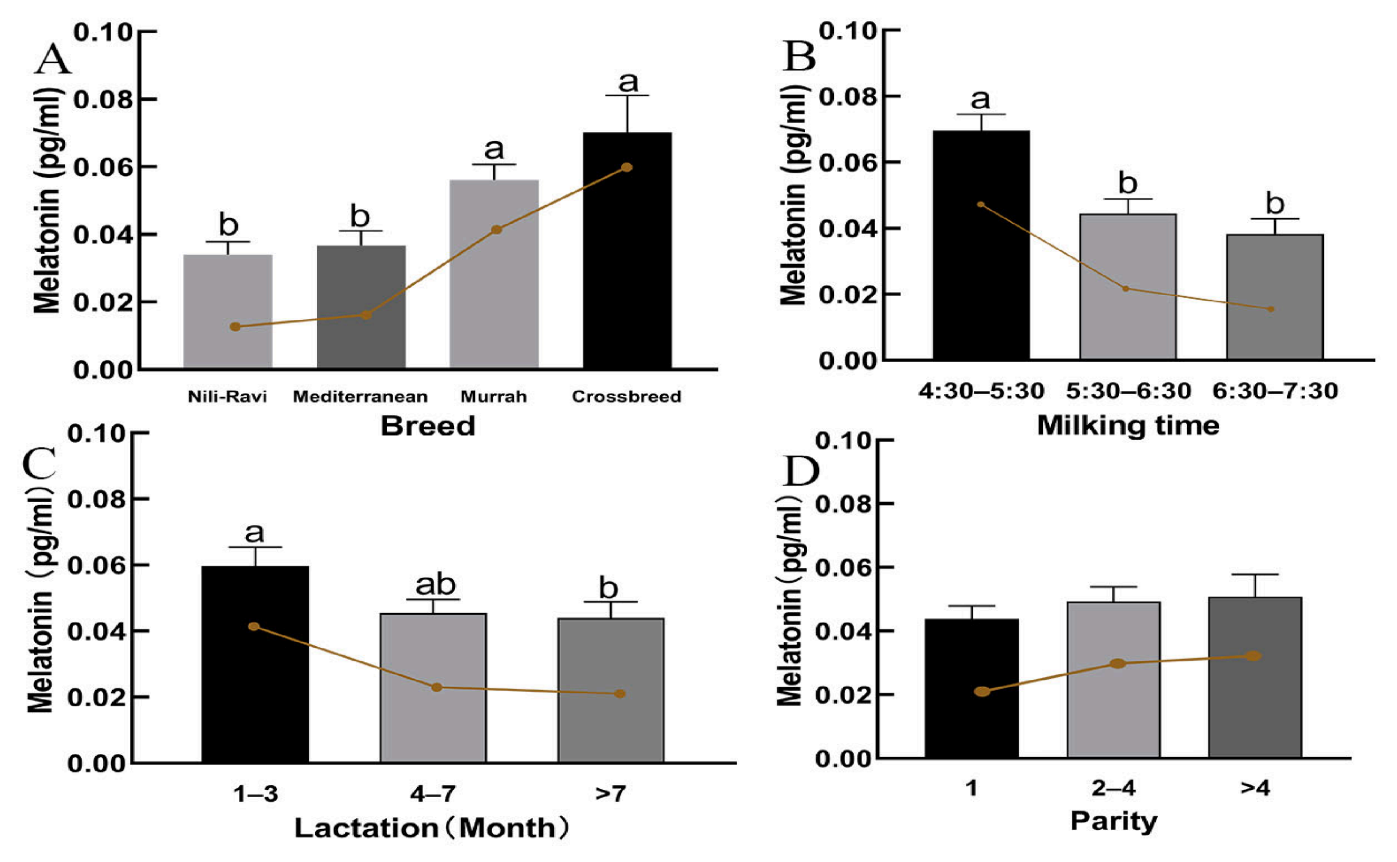
The significant relationship between the content of MT in buffalo milk and the associated factors and the trend of change. Note: (**A**) The relationship between breed and MT content; (**B**) The relationship between milking time and MT content; (**C**) The relationship between lactation and MT content; (**D**) The relationship between parity and MT content. Different lowercase letters (a, b) indicate significant differences between groups at *p* < 0.05; groups sharing at least one common letter are not significantly different; the absence of letters indicates no significant differences.

Melatonin is greatly affected by environmental factors. This study focused on the effects of milking-related factors. Based on the comparisons of the estimated marginal means (EMMs) from the generalized linear model correction, the effects of milking time, parity, breed, and lactation on melatonin content in buffalo milk were analyzed. As shown in Table 1 and Figure 2, there was a significant difference in the content of melatonin in milk among different varieties of milk (*p* < 0.05), with an overall trend: Nili-Ravi < Mediterranean < Murrah < Crossbred (Figure 2A). Among them, the content in the milk of Murrah and Crossbred was significantly higher than that in Nili-Ravi and Mediterranean *(p* < 0.05). Milking time was identified as another key factor associated with milk MT content (*p* < 0.05) (Figure 2B), with an overall decreasing trend: 4:30–5:30 am > 5:30–6:30 am > 6:30–7:30 am. The content of melatonin in milk samples collected at 4:30–5:30 am was the highest, which was significantly higher than that at 5:30–6:30 am and 6:30–7:30 am (*p* < 0.05). There was no significant difference between the 5:30–6:30 am and 6:30–7:30 am (*p* > 0.05) samples.

In addition, lactation stages were significantly associated with buffalo milk MT content (*p* < 0.05) (Figure 2C). Melatonin content in milk from buffalo in early lactation (1–3 months) was significantly higher than in late (>7 months) lactation (*p* < 0.05), and there was no significant relationship with the middle (4–7 months) (*p* > 0.05).

In terms of parity, no significant intergroup differences in buffalo milk MT content were detected among buffaloes with different parities (*p* > 0.05) (Figure 2D), and no regular variational trend was found across all parity groups.

### 2.3. Population Structure

**Figure 3:**
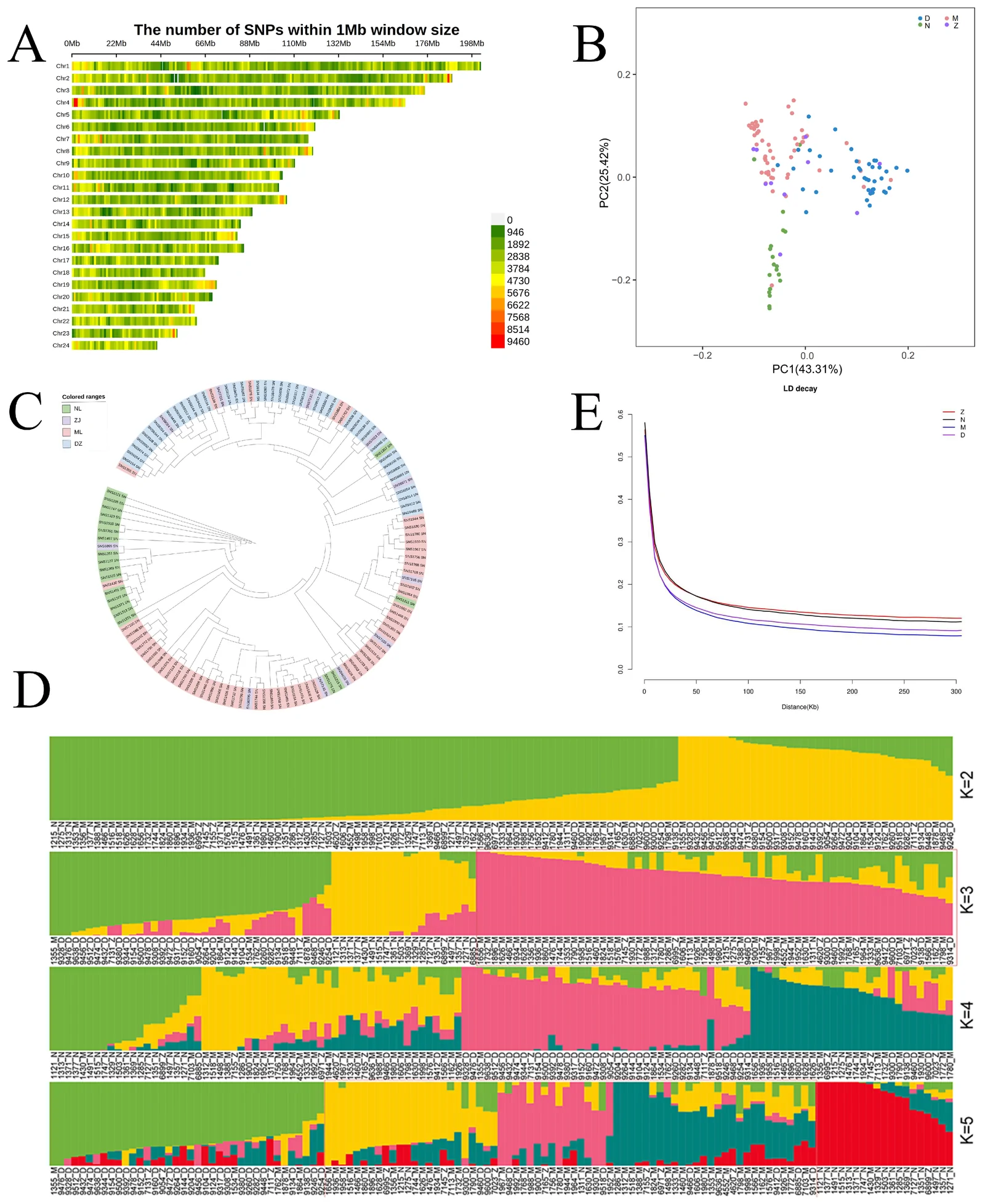
Population genetic structure based on 125 buffaloes. (**A**) SNP density plot (the color gradient from green to red indicates increasing SNP density within 1 Mb intervals); (**B**) PCA plot of the two principal components of all individuals; (**C**) SNP-based Neighbor-joining (NJ) phylogenetic tree. (**D**) Population stratification diagram with K = 2–5; (E) LD decay plot (X-axis represents physical distance, Y-axis represents LD Value (r^2^)). Note: (**A**) the x-axis represents the physical distance along each chromosome, split into 1 Mb windows; the y-axis indicates individual chromosomes, where the different colors correspond to the number of SNPs per window. (**B**,**C**) NL (green) represents Nili-Ravi population, ZJ (purple) represents Crossbred population, ML (red) represents Murrah population, and DZ (blue) represents Mediterranean population. (**D**) Population genetic structure inferred from ADMIXTURE analysis. Each vertical bar represents one buffalo individual; different colors represent different hypothesized ancestral genetic components.

Population structure is also an important endogenous factor affecting quantitative traits, so this study further analyzed the genetic structure of the buffalo population. Following quality control, a total of 11,773,341 high-quality SNPs were identified for subsequent analysis. The SNP density map shows (Figure 3A) that SNPs are distributed relatively evenly across the chromosomes, with an average of 490,555 SNPs per chromosome, and the density was more than 4.53 SNPs/Kb. Based on the genome-wide SNPs, principal component analysis (PCA, Figure 3B) and NJ phylogenetic tree construction (Figure 3C) were performed on 125 buffalo individuals. The PCA results show that PC1 had 43.31% of the genetic variance, while PC2 had 25.42% of the genetic variance, with a combined contribution of 68.73%, effectively reflecting the genetic differentiation between populations. The results of PCA and NJ tree were basically the same, and the samples could be divided into three main genetic groups as a whole: Nili-Ravi (N) formed an independent cluster, which was significantly separated from Murrah (M) and Mediterranean (D) populations. This indicates that the Nili-Ravi population had a relatively independent genetic background. Although there was a small amount of individual overlap between Murrah (M) and Mediterranean (D), they still exhibit relatively independent clustering characteristics. The individuals of Crossbred buffalo (Z) were dispersed among different groups and did not form an independent branch, which was consistent with the characteristics of the mixed genetic background of multi-parent hybrid offspring.

The results of ADMIXTURE analysis (Figure 3D) indicate that K = 5 was the expected optimal number of clusters. When K = 3, the number of clusters is more consistent with the results from PCA and the NJ tree. Comparing the population structure of different K values, it was found that when K = 5, in addition to retaining the independent genetic components of Nili-Ravi and Mediterranean buffalo, three subgroups were further differentiated within the Murrah population. The genetic structure characteristics of the remaining populations were consistent with the results of K = 3, and the mixed hybrid buffalo individuals were distributed in different populations.

Linkage disequilibrium (LD) (Figure 3E) analysis revealed obvious differentiation in LD decay levels among different buffalo populations. The LD level of the Crossbred (Z) population was the highest, followed by the Nili-Ravi (N) and Mediterranean (D) populations, while the Murrah (M) population had the lowest level. In terms of decay rate, the LD decay rate of Murrah was the fastest, which reflected that genomic recombination events in this population were more frequent, and its genetic diversity was higher. Compared with other populations, the Crossbred (Z) population may have experienced selection pressure, resulting in a relatively high degree of linkage disequilibrium.

### 2.4. Analysis of the Correlation Between AANAT and MT Content

*AANAT* is a key rate-limiting enzyme gene for MT synthesis, which regulates the production of MT in mammals. A total of eight SNPs were screened within the *AANAT* region. Table 2 and Table 3 summarize the genotype frequency, allele frequency, *H*o, *H*e, and HWE of the eight SNPs in the buffalo *AANAT* gene. The results show that SNP2 and SNP8 deviated from Hardy–Weinberg equilibrium, while no homozygous mutant genotype was detected in SNP1. The wild-type allele frequency of all SNPs was higher than the mutant allele frequency.

**Figure 4:**
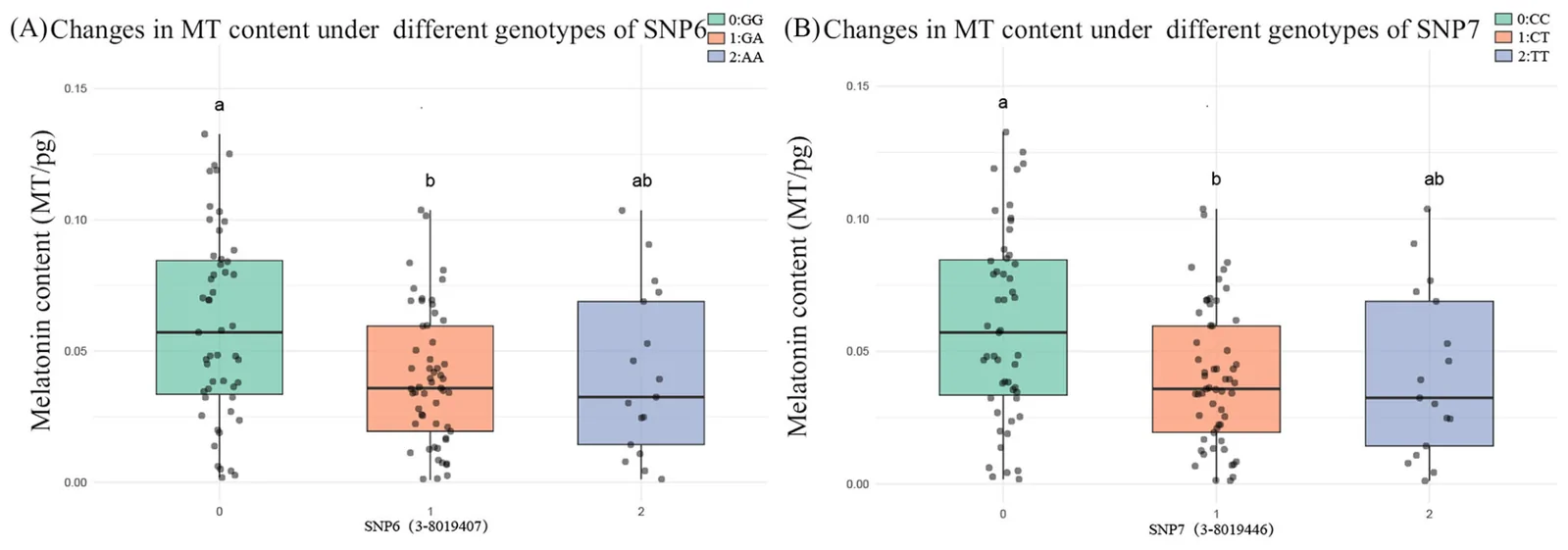
Changes in MT content in buffalo milk under different genotypes of *AANAT.* Note: The results shown in this figure represent genotypes that are significant in GLM-EMM and also significant in Kruskal–Wallis/Dunn’s tests. Different lowercase letters (a, b) indicate significant differences between groups at *p* < 0.05; groups sharing at least one common letter are not significantly different.

Association analysis between SNPs and MT concentration revealed that two SNPs were significantly correlated with MT synthesis (*p* < 0.05), which were 3-8019407 (*p* = 0.03048) and 3-8019446 (*p* = 0.03048) (Table 4). The two SNPs were in the intron region, and the significant locus 3-8019407 was completely linked to 3-8019446 (R^2^ = 1.0). Post hoc pairwise comparisons of the estimated marginal means (EMMs) were performed using the least significant difference (LSD) test; the MT secretion level of buffalo milk with homozygous wild genotype GG (57.98 pg/mL) of 3-8019407 was significantly higher than that of GA (39.11 pg/mL) (*p* < 0.05) (Figure 4A). The level of MT secretion in buffalo milk of homozygous wild genotype CC (57.98 pg/mL) of 3-8019446 was significantly higher than that of CT (39.11 pg/mL) (*p* < 0.05) (Figure 4B), while GG (CC) and AA (TT), GA (CT) and AA (TT) did not reach a significant level.

**Table 4:** Association information of 2 SNPs with milk MT secretion content.

Gene	*AANAT* (Chromosome 3: 8,018,937–8,020,953)					
>Site	SNP6 (3-8019407) *p* = 0.0305			SNP7 (3-8019446) *p* = 0.0305		
Genotype	GG (*N* = 51)	GA (*N* = 57)	AA (*N* = 17)	CC (*N* = 51)	CT (*N* = 57)	TT (*N* = 17)
Melatonin (pg/mL) EMM ± SEM Mean ± SEM	57.98 ± 5.37 59.54 ± 5.01	39.11 ± 3.44 39.12 ± 3.40	41.43 ± 6.74 41.26 ± 7.66	57.98 ± 5.37 59.54 ± 5.01	39.11 ± 3.44 39.12 ± 3.40	41.43 ± 6.74 41.26 ± 7.66

Abbreviations: *N*, number of samples. *p*, *p*-Value of the association between SNP genotype and phenotype, representative nominal *p*-Value. EMM ± SEM, the estimated marginal mean ± the standard error of the mean. Mean ± SEM, arithmetic mean ± the standard error of the mean.

### 2.5. Verification of SNPs

**Figure 5:**
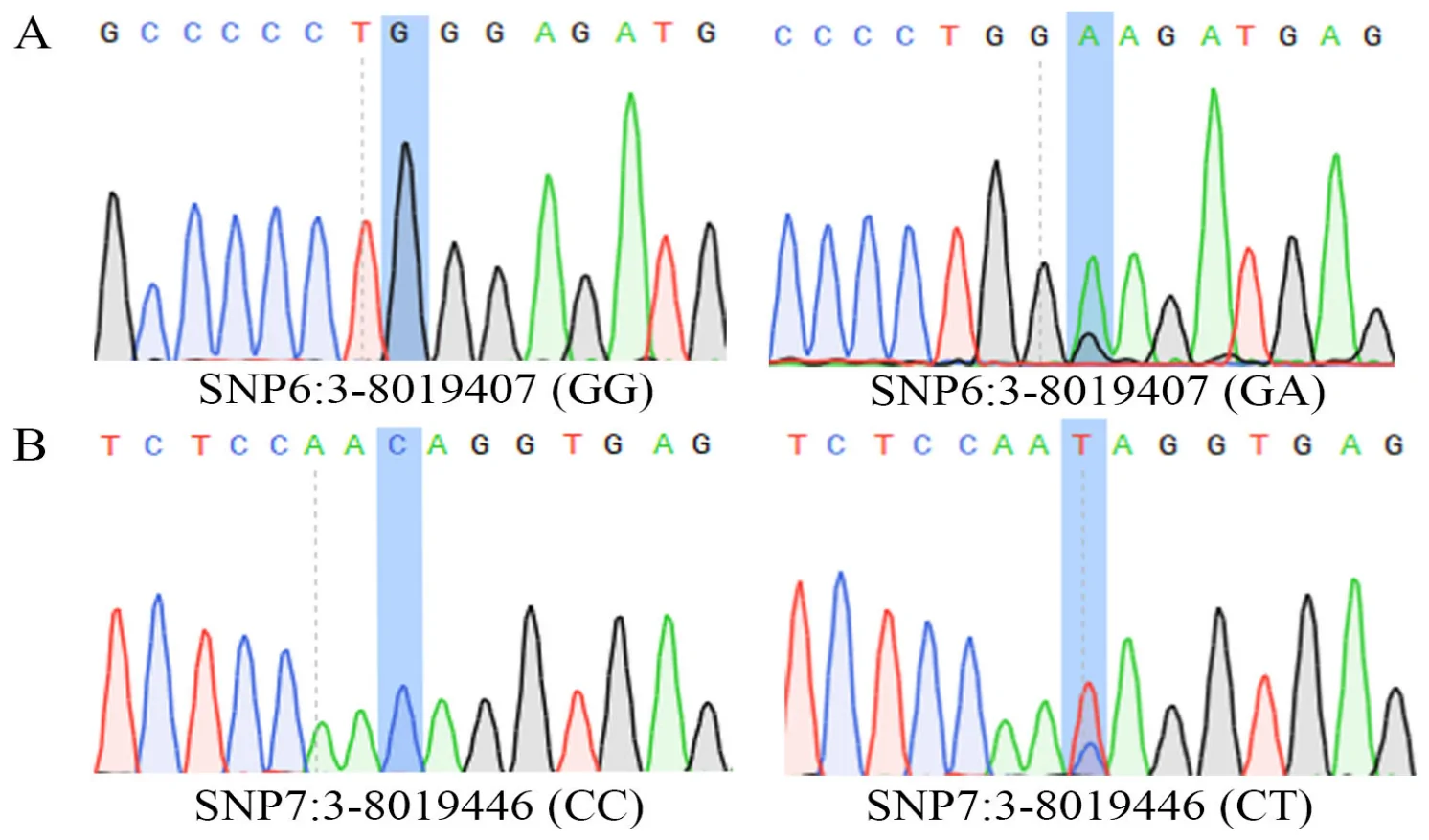
Genotypic sequencing peak maps. (**A**) Genotypes of 3-8019407; (**B**) genotypes of 3-8019446.

Based on the above findings, subsequent validation analyses focused on the high-melatonin GG (CC) genotype and the low-melatonin GA (CT) genotype. The SNPs obtained from 56 experimental buffalo individuals were validated (56 buffalo individuals, one sample per individual), as shown in Figure 5. The genotypes, including GG and GA at SNP6 (Figure 5A), as well as CC and CT at SNP7 (Figure 5B), were detected in these validation samples. Combined with the association analysis data in Table 5, post hoc pairwise comparisons of estimated marginal means (EMMs) revealed that the MT content of wild homozygous genotype GG (CC) was significantly higher than that of heterozygous genotype GA (CT) (*p* < 0.05).

## 3. Discussion

MT is one of the important functional active substances in milk, which plays an important role in enhancing the nutritional value of dairy products, promoting sleep, and improving antioxidant capacity [27]. MT exhibits a typical circadian secretion pattern, where its synthesis and release are regulated by photoperiod. Higher MT levels are observed in milk collected under nighttime or low-light conditions [28]. Studies have found that in addition to external factors such as light exposure, the secretion and synthesis of MT may also be regulated by genetic factors, and substantial individual differences exist in MT synthesis capacity [23]. This study carried out quantitative analysis of MT content in buffalo milk collected from the same ranch. The results showed that the concentration distribution range was 0.80–132.67 pg/mL. Among the buffalo milk samples, 91.2% of buffalo milk melatonin concentrations were lower than 100 pg/mL, and only 8.8% of buffalo milk melatonin contents were higher than 100 pg/mL. Significant individual variation was observed, indicating the presence of naturally high-yield MT individuals within the buffalo population. This provides an important basis for the subsequent identification of genetic regulatory factors affecting MT content in milk and the development of functional dairy products.

We further investigated the effects of breed, milking time, lactation, and parity on melatonin content in buffalo milk. Correlation analysis between the MT content in milk and these four factors revealed that breed, milking time, and lactation were significantly associated with MT content. Among these factors, milking time had the most significant effect on MT content, with the highest MT content observed at 4:30–5:30 am, with a gradual decline during 5:30–6:30 am and 6:30–7:30 am, indicating that the MT content in buffalo milk had an obvious temporal rhythm. This is consistent with the general patterns of photoperiodic regulation of MT in other mammals [29,30]. MT is mainly synthesized and secreted under dark conditions. Light can inhibit *AANAT* activity through the retinal-suprachiasmatic nucleus-pineal neuroendocrine pathway, thereby reducing the level of MT synthesis [9,31,32]. Numerous studies have confirmed that *AANAT* expression and enzyme activity exhibit significant circadian rhythms. Its expression typically increases during the dark phase and is suppressed under light conditions. Night-time light exposure can rapidly reduce *AANAT* mRNA, protein levels, and enzyme activity, thereby inhibiting MT synthesis [10,32,33]. Studies have shown that photoperiod and light intensity not only affect the level of MT in plasma or milk but also affect milk yield, milk composition, and endocrine indicators [12]. In this study, the MT level of milk samples at 4:30–5:30 am was the highest, indicating that the early morning dark period may be the key time window for obtaining high-MT buffalo milk. It also suggests that the milking time, light intensity and dark preservation should be regarded as important production control factors in the future development of high-MT buffalo milk. In addition, the study found that MT content in milk was significantly higher during the early lactation period (1–3 months) than during the late lactation periods (≥7 months), which may be related to oxidative stress and transport regulation. During the early lactation, the mammary gland of cows exhibits enhanced transport capacity for substances such as glucose, lipids, and calcium, accompanied by obvious energy metabolism and immune adaptation [34,35]. The increased secretion of MT may be an adaptive regulation of the body and teat tissue to alleviate oxidative stress because MT has antioxidant and immunomodulatory effects [36].

In addition, buffalo breeds also affect the differences in milk MT content. MT content in Crossbred buffalo and Murrah buffalo milks was significantly higher than in Nili-Ravi buffalo and Mediterranean buffalo milks, indicating that different genetic backgrounds may influence the capacity of MT synthesis, transport, or secretion in the mammary gland. This is in agreement with a study that reported that there were differences in hormone secretion levels among different breeds (genetic background) [37]. Population structure analysis revealed that the 125 buffaloes could be assigned to distinct genetic groups. The results of PCA, NJ tree, and ADMIXTURE analysis were basically consistent, indicating that there was indeed genetic differentiation in this study population. When analyzing the relationship between SNPs and milk MT content, this study included breed, milking time, lactation stage, and parity in the model to reduce confounding effects caused by environmental factors.

*AANAT* represents the primary candidate gene of interest in this study. Tryptophan acts as the starting precursor within the canonical MT biosynthetic pathway. It is metabolized through 5-hydroxytryptamine (serotonin) to yield N-acetylserotonin, which is subsequently converted into MT under the catalysis of *ASMT*. Since the N-acetylation reaction of 5-hydroxytryptamine is mediated by the *AANAT*-encoded enzyme, *AANAT* is recognized as a pivotal enzyme governing melatonin biosynthesis [38]. *AANAT* is not only involved in MT synthesis in the pineal gland but is also expressed in a variety of peripheral tissues and is closely related to local MT synthesis and physiological regulation [39,40]. Animal studies have shown that decreased expression or activity of *AANAT* can lead to decreased MT content [41]. In goats, long-term dexamethasone (Dex) treatment downregulates the expression of *AANAT* in the pineal gland and colon and disrupts the MT rhythm, indicating that *AANAT* plays an important role in the MT synthesis rhythm of ruminants [42]. These studies support the rationality of *AANAT* as a candidate gene for buffalo milk MT content traits.

In this study, a total of eight SNPs were screened in the buffalo *AANAT* region. Among them, 3-8019407 and 3-8019446 were significantly correlated with the MT content in buffalo milk, and the two loci were completely linked (R^2^ = 1.0), indicating that these two SNPs were associated with the same signal. Individuals carrying the GG genotype at 3-8019407 and the CC genotype at 3-8019446 exhibited a buffalo milk MT content of 57.98 pg/mL, which was significantly higher than that of GA/CT genotype individuals. No significant difference was observed between GG/CC and AA/TT genotypes. Further verification also showed that the MT content of wild homozygous individuals was significantly higher than that of heterozygous individuals. In the verification population, the MT content was 72.87 pg/mL for wild-homozygous genotypes and 50.24 pg/mL for heterozygous genotypes. This suggests that heterozygous disadvantage may occur at a specific site within the *AANAT* gene [43]. It is worth noting that these two SNPs reside in the intron region. Although the intron SNPs do not directly alter the amino acid sequence of a protein, intron variants can still regulate gene expression by affecting cis-regulatory element activity, transcription factor binding, chromatin accessibility or pre-mRNA splicing [44,45,46]. Therefore, the SNPs identified within the *AANAT* region in this study may be associated with MT content in buffalo milk by regulating *AANAT* expression. In the present study, we were unable to elucidate the exact mechanism by which SNPs in *AANAT* relate to MT content in buffalo milk. However, these SNPs show an association with MT content, and elucidating the molecular mechanism underlying this relationship is a goal for future research.

## 4. Materials and Methods

### 4.1. Sample Collection and Extraction

Experimental samples were collected in May 2025 from the buffalo farm of the Buffalo Research Institute, Chinese Academy of Agricultural Sciences and Guangxi Zhuang Nationality Autonomous Region. All lactating buffaloes were maintained under identical feeding and management conditions. The experimental buffaloes were housed in open pens. They were fed a total mixed ration (TMR) formulated by the breeding farm twice daily (at 8:00 am and 4:00 pm). Water was available ad libitum throughout the experiment, and feed quality remained constant during the sampling period. In accordance with routine farm production practices, milking was conducted between 4:30 and 7:30 am (sunrise and the beginning of civil twilight occurred at 6:08 am and 5:44 am). A total of 125 buffalo milk samples (125 buffalo individuals, one sample per individual) were collected, and all collected samples were whole milk (lactation months 1–14). Information on parity, breed, lactation, and milking time was recorded according to farm production records and the order of sample collection. This procedure involved manual milking. Before milking, teats were cleaned and disinfected with a disposable towel dipped in povidone-iodine solution. When milking, the first three streams of milk were discarded, and milk samples were collected into 50 mL light-protected sterile centrifuge tubes. The samples were placed in ice, transported to the laboratory immediately after collection, and stored at −80 °C until further analysis.

Melatonin extraction from buffalo milk was performed with reference to T/BDAS 001-2023, “Determination of Melatonin content in milk—Liquid chromatography–tandem mass spectrometry” with partial modifications. The LC-MS/MS detection was carried out on a SCIEX Triple Quad 6500+ liquid chromatography–tandem mass spectrometry (LC-MS/MS) system (SCIEX, Framingham, MA, USA).

Frozen milk samples were thawed at room temperature, sonicated in an ultrasonic cleaner at 37 °C for 30 min (CH03BM, Suzhou Chuanghui Electronics Co., Ltd., Suzhou, Jiangsu, China), and centrifuged at 4000 rpm (Eppendorf 5804R, Eppendorf SE, Hamburg, Germany) and 4 °C for 5 min for the preliminary defatting process. Approximately 1 mL of each sample was accurately transferred into a 15 mL centrifuge tube. Then, 10 μL of deuterated melatonin standard working solution (10 μg/mL) and 4 mL of methanol pre-cooled to −20 °C were added. The mixture was vortexed for 5 min and kept at −18 °C for 30 min. The samples were centrifuged at 12,000 rpm and 4 °C for 5 min using a refrigerated high-speed centrifuge (Thermo Heraeus Mnltifuge X1R, Thermo Fisher Scientific, Waltham, MA, USA). The supernatant was collected and centrifuged again under the same conditions.

The supernatant was evaporated to dryness under a nitrogen stream in a 50 °C water (MultiVap-8, Beijing LabTech Instruments Co., Ltd, Beijing, China) and reconstituted in 1 mL of water. HLB solid-phase extraction cartridges were activated sequentially with 1 mL of methanol and 1 mL of water. The solution was loaded onto the HLB at a flow rate of approximately one drop per second. The solution was passed through the HLB at one drop per second. The HLB was washed with 1 mL of 5% methanol in water after vacuum drying, then eluted with 1 mL of methanol. The eluate was collected, filtered through a 0.22 µm membrane filter, and transferred into an amber volumetric flask for subsequent LC–MS/MS analysis. All procedures were performed under light-protected conditions using aluminum foil.

Liquid chromatography conditions: The sample was analyzed using liquid chromatography–tandem mass spectrometry. The chromatographic column was C18-AQ (2.2 mm × 100 mm, 1.8 µm) (Agilent, Santa Clara, CA, USA). A rapid gradient elution was performed for 9 min. The mobile phase was as follows: Phase A was 0.1% formic acid aqueous solution, and Phase B was methanol with a flow rate of 0.25 mL/min. The column temperature was 40 °C, while the injection volume was 10 μL.

MS analysis was performed using electrospray ionization (ESI). Data were acquired in positive-ion mode under multiple reaction monitoring (MRM). The spray voltage was set at 5500 V, and the ion source temperature was maintained at 450 °C. The sheath gas flow rate was 11 L/min, and nitrogen was used as the auxiliary gas at a flow rate of 6 L/min. Argon served as the collision gas at 0.2 Pa. Qualitative ion pairs, quantitative ion pairs and collision energy were determined according to reference values. The relevant conditions are as shown in Table 6.

### 4.2. Genotyping and Population Structure Analysis

SNP Calling was performed on the buffalo population using the buffalo genome (GCF_019923935.1) as the reference genome, and the distribution and density of high-quality SNPs in the genome were visualized using the CMplot package. Principal component analysis (PCA) was performed using PLINK v1.90-b.8 software, and then the distance matrix of 125 individuals was constructed based on high-quality SNP data using VCF2Dis v1.50 (https://github.com/BGI-shenzhen/VCF2Dis (accessed on 24 April 2026)) software. The NJ phylogenetic tree was constructed using FastME and visualized through the iTOL online platform (https://itol.embl.de/ (accessed on 24 April 2026)). ADMIXTURE v1.3.0 software was used to infer the population structure of buffalo individuals. The range of tested K values was set to 1 to 6, and the optimal K value was determined based on the lowest cross-validation error. Pairwise linkage disequilibrium (LD, r^2^) between SNPs was calculated by PopLDdecay v1.4.1 software (https://github.com/hewm2008/PopLDdecay (accessed on 11 May 2026)), and the LD decay curve was generated.

### 4.3. Statistical Analysis

Melatonin content was a positive continuous variable and showed a skewed distribution. The model was set to a Gamma distribution, and a log link function was used. The model parameters were estimated by the maximum likelihood method, and the overall significance of the fixed effect was estimated by Type III Wald. The specific models are as follows:

*Y_ijkl_* ~ Gamma(*μ_ijkl_,ϕ*)

g(*μ_ijkl_*) = log(*μ_ijkl_*) = *β_0_* + *A_i_* + *B_j_* + *C_k_* + *D_l_*

In the formula, Y*_ijkl_* is the expected value of MT content under the *i*~(th) lactation, the *j*~(th) breeds, the *k*~(th) milking time, and the *l*~(th) parity. *ϕ* is the dispersion (or scale) parameter of the Gamma distribution. *μ_ijkl_* is the corresponding expected MT content, and *β_0_* is the intercept; *A_i_* is the fixed effect of lactation; *B_j_* is the fixed effect of breeds; *C_k_* is the fixed effect of milking time; and *D_l_* is the fixed effect of parity. The lactation month was divided into three stages: 1–3 months as the first stage, 4–7 months as the second stage and >7 months as the third stage. Model parameters were estimated using the maximum likelihood method, the significance of the effect was tested by Type III Wald chi-square test, and Pairwise comparisons between groups were performed using the Estimated Marginal Means (EMM) method. *p* > 0.05 was not significant.

Genotype and allele frequencies, observed heterozygosity (*H*o) and expected heterozygosity (*H*e) were calculated using PLINK v1.90-b.8 software. The allele frequency of each SNP was calculated to assess deviation from Hardy–Weinberg equilibrium (HWE). SNP–phenotype correlations were analyzed using R v4.3.2 software. The following statistical models were established based on milking time, parity, lactation, and breed:

g(*μ_eijkl_*) = log(*μ_eijkl_*) = *β_0_* + *SNP_e_* + *A_i_* + *B_j_* + *C_k_* + *D_l_*

In the formula, *μ_eijkl_* is the expected value of melatonin content under the *e*~(th) SNP genotype; the *j*~(th) breeds, the *k*~(th) milking time, and the *l*~(th) parity.

*β*_0_ is the intercept (expected value); *SNP_e_* is the effect of SNP genotype; *A_i_* is the fixed effect of lactation; *B_j_* is the fixed effect of breeds; *C_k_* is the fixed effect of milking time; and *D_l_* is the fixed effect of parity. Analysis of the correlation between SNPs and MT content; *p* < 0.05 indicates a significant difference, while *p* > 0.05 indicates no significant difference. For each SNP, the genotypes were grouped (0/1/2); a Gamma generalized linear model with a log link function was fitted to account for the fixed effects of lactation stage, breed, milking time and parity; estimated marginal means (EMMs) were calculated; and the post hoc multiple comparisons of EMMs were performed using the LSD test. The Kruskal–Wallis test was used to compare the overall difference in the median MT content between the groups. When the Kruskal–Wallis test was significant (*p* < 0.05), Dunn’s test was used to compare differences in MT content among different genotypes. Plots were generated using R v4.3.2 software.

### 4.4. SNP Validation

SNPs were identified in Section 4.3. Buffalo with different mutations at the SNPs with the highest correlation were selected. A total of 56 newly collected buffalo milk samples (56 buffalo individuals, one sample per individual) were used as the validation cohort. Using Sanger sequencing and resequencing data, we identified the *AANAT* genotypes associated with high and low MT content in buffalo. MT content was measured in 56 buffalo milk samples carrying the relevant mutations to verify the association between different genotypes and MT content.

The DNA extraction was conducted using a Cell Genomic DNA Extraction Kit (DP304, Tiangen Biotech (Beijing) Co., Ltd., Beijing, China) to extract genomic DNA from buffalo milk. DNA quality was assessed using a 1% agarose gel, and DNA purity and concentration were measured using a NanoDrop 2000 spectrophotometer (Thermo NanoDrop 2000, Thermo Fisher Scientific, Waltham, MA, USA). The samples were diluted to 50 ng·μL^−1^ and stored at −20 °C.

Primer Design: Primers for *AANAT*-related SNPs were designed using Primer3 (Table 7) and sent to Sangon Biotech (Shanghai) Co., Ltd. for synthesis.

PCR Amplification: PCR amplification was performed using 2× Tag Plus Master Mix II P213-02 (Vazyme; Nanjing, China). The PCR reaction mixture (25 μL) consisted of: 12.5 μL 2× Taq Master Mix, 1 μL upstream primer (10 μmol·L^−1^), 1 μL downstream primer (10 μmol·L^−1^), 2 μL DNA template, and 8.5 μL ddH_2_O. The reaction conditions were as follows: 3 min of pre-denaturation at 95 °C, followed by 30 cycles consisting of 15 s denaturation at 95 °C, 15 s annealing at 56–60 °C, and 45 s extension at 72 °C, then a final extension at 72 °C for 5 min, and the products were stored at 4 °C.

Genotyping: The products were sent to Sangon Biotech (Shanghai Sangon) Co., Ltd. for Sanger sequencing (ABI 3730 automated sequencer, Thermo Fisher Scientific, Waltham, MA, USA), and the genotyping results were interpreted using SnapGene v8.0 software. Some re-sequenced samples were interpreted directly from the genotype files. SPSS 21.0 software was used to analyze the correlation between the two genotypes. Statistical analyses were performed using the same analytical approaches described in Section 4.3, with breed, lactation, and parity included as covariates to account for their potential effects.

## 5. Conclusions

In this study, the content of melatonin (MT) in milk was determined by LC-MS/MS based on 125 buffalo milk samples. The results showed that the content of MT in milk was significantly different among buffalo individuals, suggesting that there were naturally high-MT individuals in the buffalo population. The MT content in milk was significantly associated with breed, milking time, and lactation period, but not with parity. Among them, the milk samples collected earlier in the morning and pre-lactation milk samples had higher MT content. At the same time, eight SNPs were identified in the *AANAT* region by WGS analysis. Among them, intron sites 3-8019407 and 3-8019446 were significantly correlated with MT content in milk. Individuals carrying the wild homozygous genotypes GG and CC presented significantly higher milk MT levels than their corresponding heterozygous counterparts, and this association was further validated in the verification population. The above results indicate that *AANAT* genetic variation may be involved in the regulation of MT content in buffalo milk. These results indicate that the early-morning milking period and the early-lactation can serve as critical windows for obtaining high-MT milk. Furthermore, genomic variations in the *AANAT* gene may also represent important genetic factors regulating MT content in buffalo milk. The results provide a basis for the selection of high-MT milk 430 buffalo germplasm and the development of functional buffalo milk. Future research should further explore the molecular mechanisms by which these intronic SNPs modulate *AANAT* gene expression and should increase sample sizes to evaluate the practical application value of these two SNP markers in water buffalo breeding programs.

## Figures and Tables

**Table 1:** Relationships between melatonin content and various factors in milk.

Independent Variable	Χ^2^	df	*p*-Value
Milking time	22.016	2	*p* < 0.05
Breed	15.757	3	*p* < 0.05
Lactation	7.612	2	*p* < 0.05
Parity	1.384	2	*p* > 0.05

Abbreviations: Χ^2^, chi-square statistic. df, degrees of freedom; *p*-Value, *p*-Value obtained from associations between each independent variable and milk melatonin content.

**Table 2:** SNPs located within the genomic region of the *AANAT*.

SNP	Associated Gene	Chromosome	Locus	Ref	Alt
SNP1	gene-*AANAT*	3	8019065	G	T
SNP2	gene-*AANAT*	3	8019124	C	T
SNP3	gene-*AANAT*	3	8019142	G	A
SNP4	gene-*AANAT*	3	8019171	G	A
SNP5	gene-*AANAT*	3	8019215	G	A
SNP6	gene-*AANAT*	3	8019407	G	A
SNP7	gene-*AANAT*	3	8019446	C	T
SNP8	gene-*AANAT*	3	8019819	C	T

Abbreviations: Ref, the nucleotide at this locus in the buffalo reference genome; Alt, the alternative nucleotide detected in these buffalo individuals.

**Table 3:** Genetic diversity of the *AANAT*.

SNP	Genotype Frequency			Allele Frequency		Genetic Parameters		
	D	H	R	Ref	Mut	*H*o	*H*e	HWE (*p*-Value)
SNP1 (3-8019065)	123	2	0	0.992	0.008	0.016	0.016	1
SNP2 (3-8019124)	109	13	3	0.924	0.076	0.104	0.140	0.0211
SNP3 (3-8019142)	93	29	3	0.860	0.140	0.232	0.241	0.7069
SNP4 (3-8019171)	93	29	3	0.860	0.140	0.232	0.241	0.7069
SNP5 (3-8019215)	109	16	0	0.936	0.064	0.128	0.120	1
SNP6 (3-8019407)	51	57	17	0.636	0.364	0.456	0.463	0.8485
SNP7 (3-8019446)	51	57	17	0.636	0.364	0.456	0.463	0.8485
SNP8 (3-8019819)	109	13	3	0.924	0.076	0.104	0.140	0.0211

Abbreviations: SNP, Single-nucleotide polymorphism; Genotype frequency, Genotypic frequency of corresponding genotype; Allele frequency, Allelic frequency of corresponding allele; D, Dominant genotype frequency; H, Heterozygous genotype frequency; R, Recessive genotype frequency; Ref, Reference allele; Mut, Mutant allele; *H*o: Observed heterozygosity; *H*e, Expected heterozygosity; HWE (*p*-Value), *p*-Value of Hardy–Weinberg equilibrium test.

**Table 5:** Association between different genotypes of SNPs and MT content.

Gene	*AANAT* (Chromosome 3: 8,018,937–8,020,953)			
Site	SNP6 (3-8019407) (*p* < 0.05)		SNP7 (3-8019446) (*p* < 0.05)	
Genotype	GG (*N* = 28)	GA (*N* = 28)	CC (*N* = 28)	CT (*N* = 28)
Melatonin (pg/mL) EMM ± SEM Mean ± SEM	72.87 ± 6.44 71.68 ± 4.73	50.24 ± 4.44 51.64 ± 4.38	72.87 ± 6.44 71.68 ± 4.73	50.24 ± 4.44 51.64 ± 4.38

Abbreviations: N, number of samples. *p*, *p*-Value of the association between genotype and phenotype; indicated after post hoc pairwise comparisons of estimated marginal means (EMMs) using the least significant difference (LSD) test, the melatonin content differed significantly between the two genotypes. EMM ± SEM, the estimated marginal mean ± the standard error of the mean. Mean ± SEM, arithmetic mean ± the standard error of the mean.

**Table 6:** Reference values for qualitative and quantitative ion pairs and collision energies.

Compound	Qualitative Ion Pairs (m/z)	Quantitative Ion Pairs (m/z)	DP (V)	CE (V)
Melatonin	233.1 > 174.2	233.1 > 174.2	70	23
	233.1 > 159.1		70	35
Melatonin-D4	237.1 > 178.2	237.1 > 178.2	70	23

Abbreviations: DP, declustering potential. CE, collision energy.

**Table 7:** Sequence list of validation primers.

Primers	Gene	Primers Sequence (5′–3′)	Size (bp)
SNP6&7	*AANAT*	F:CCGCCCCTAAGAACTTCACA	586
		R:CACGCACAAAACACACCGTCA	

## Data Availability

The variation data reported in this paper have been deposited in the Genome Variation Map (GVM) in the National Genomics Data Center, Beijing Institute of Genomics, Chinese Academy of Sciences and China National Center for Bioinformation, under accession number GVM001598. Other supporting raw data are available as Supplementary Materials in this MDPI submission.

## Supplementary Materials

The following supporting information can be downloaded at: https://www.mdpi.com/article/10.3390/ijms27209178/s1.
